# A Retrospective and Multicenter Study on COVID-19 in Inner Mongolia: Evaluating the Influence of Sampling Locations on Nucleic Acid Test and the Dynamics of Clinical and Prognostic Indexes

**DOI:** 10.3389/fmed.2022.830484

**Published:** 2022-03-30

**Authors:** Lan Yu, Ailan Wang, Tianbao Li, Wen Jin, Geng Tian, Chunmei Yun, Fei Gao, Xiuzhen Fan, Huimin Wang, Huajun Zhang, Dejun Sun

**Affiliations:** ^1^Inner Mongolia Key Laboratory of Gene Regulation of the Metabolic Diseases, Clinical Medical Research Center, Inner Mongolia People’s Hospital, Hohhot, China; ^2^Endocrinology Department, Inner Mongolia People’s Hospital, Hohhot, China; ^3^Geneis (Beijing) Co., Ltd., Beijing, China; ^4^Qingdao Geneis Institute of Big Data Mining and Precision Medicine, Qingdao, China; ^5^Key Laboratory of National Health Commission for the Diagnosis and Treatment of COPD, Department of Pulmonary and Critical Care Medicine, Inner Mongolia People’s Hospital, Hohhot, China; ^6^Department of Pulmonary and Critical Care Medicine, The Fourth Hospital of Inner Mongolia, Hohhot, China; ^7^Department of Pulmonary and Critical Care Medicine, Xilin Gol League Central Hospital, Xilinhot, China; ^8^Department of Pulmonary and Critical Care Medicine, The First Affiliated Hospital of Baotou Medical College, Inner Mongolia University of Science and Technology, Baotou, China; ^9^Department of Mathematics, Shaoxing University, Shaoxing, China

**Keywords:** COVID-19, clinical characteristics, prognosis model, feces testing, machine learning

## Abstract

COVID-19 is spreading widely, and the pandemic is seriously threatening public health throughout the world. A comprehensive study on the optimal sampling types and timing for an efficient SARS-CoV-2 test has not been reported. We collected clinical information and the values of 55 biochemical indices for 237 COVID-19 patients, with 37 matched non-COVID-19 pneumonia patients and 131 healthy people in Inner Mongolia as control. In addition, the results of dynamic detection of SARS-CoV-2 using oropharynx swab, pharynx swab, and feces were collected from 197 COVID-19 patients. SARS-CoV-2 RNA positive in feces specimen was present in approximately one-third of COVID-19 patients. The positive detection rate of SARS-CoV-2 RNA in feces was significantly higher than both in the oropharynx and nasopharynx swab (*P* < 0.05) in the late period of the disease, which is not the case in the early period of the disease. There were statistically significant differences in the levels of blood LDH, CRP, platelet count, neutrophilic granulocyte count, white blood cell number, and lymphocyte count between COVID-19 and non-COVID-19 pneumonia patients. Finally, we developed and compared five machine-learning models to predict the prognosis of COVID-19 patients based on biochemical indices at disease onset and demographic characteristics. The best model achieved an area under the curve of 0.853 in the 10-fold cross-validation.

## Introduction

Currently, the world is overwhelmed by an infectious disease called coronavirus disease 2019 (COVID-19), which is caused by the severe acute respiratory syndrome coronavirus 2 (SARS-CoV-2) (1). SARS-CoV-2 is highly infectious *via* respiratory droplets, direct contact, and even fecal-oral transmission (2–4). According to the World Health Organization statement on November 10, 2021, the epidemic spread rapidly to 219 countries worldwide. There have been more than 250 million confirmed infected individuals and 5 million deaths globally, and the numbers are still increasing. Until now, it remains unknown whether SARS-CoV-2 will disappear or, in the end, become a seasonal infection.

Most COVID-19 patients will suffer from acute respiratory tract infections. In contrast, a few patients can develop novel coronavirus pneumonia, acute respiratory distress syndrome, acute respiratory failure, or other severe secondary complications (5, 6). The COVID-19 patients are clinically diagnosed as mild, moderate, or severe according to a comprehensive judgment based on epidemiological history, clinical manifestations, laboratory testing, and imaging examinations. Mild patients usually present common symptoms of respiratory system infection such as fever, cough, and fatigue, and some may have no obvious clinical symptoms (5, 7, 8). In contrast, most moderate and severe patients will present chest computed tomography (CT) with typical imaging findings, including ground-glass opacification and mixed consolidation that mostly appears at the peripheral area of both lungs (9, 10). In addition to abnormal CT, patients infected with SARS-CoV-2 also show altered hematologic parameters, including (1) significantly higher level of transaminases, lactate dehydrogenase (LDH), C-reactive protein (CRP), D-dimer, procalcitonin, interleukin-6, white blood cell count, neutrophil count, erythrocyte sedimentation rate, and ferritin; (2) lower lymphocyte count and decreased red blood cell (RBC); and (3) lower levels of immunoglobulin G and hemoglobin (11, 12).

The clinical diagnosis of COVID-19 patients and their prognosis prediction are critical for preventing and treating this highly infectious disease. According to the current diagnostic criteria, the gold standard to identify SARS-CoV-2 infections or determine discontinuation of quarantine and discharge is the nucleic acid test performed using real-time reverse transcriptase-polymerase chain reaction (RT-PCR) (13, 14). However, an essential issue with the nucleic acid detection-based approaches is the risk of eliciting false-negative results. For example, many suspected cases with typical characteristics of SARS-CoV-2 infection in CT images and clinical were diagnosed as negative by nucleic acid detection (15). There are also cases in which positive RT-PCR test results were observed during the reexamination in patients who discontinued quarantine or were discharged (16, 17). The main reason for this phenomenon is that the accuracy of RT-PCR detection depends on virus load, sample quality, quality of detection reagent, and so on (18), among which viral load kinetics in different anatomic sites and at different disease stages might be the most critical factors (19).

The optimal sampling sites at different stages of COVID-19 remain to be fully determined. Yang et al. has reported sputum as the most sensitive sample for laboratory diagnosis of SASR-CoV-2, followed by nasal swabs (20). They also recommended detecting SARS-CoV-2 in bronchoalveolar lavage fluid (BALF) to diagnose and monitor viruses in severe cases. However, BALF samples are inappropriate for routine laboratory monitoring on disease progression because of the inconvenient sampling procedure. Other specimens such as sputum, nasal swab, pharyngeal swabs, and feces are faster, simpler, and safer than BALF. Furthermore, the study did not consider the kinetic dynamics on different stages of the COVID-19 patients (21). To our best knowledge, no study has systematically compared SASR-CoV-2 detection in different sample types over time.

Here, we present a retrospective and multicenter study on COVID-19 in Inner Mongolia. We compare nucleotide acid testing among pharynx swabs, oropharynx swabs, and feces samples and at early, middle, and late stages of COVID-19. In addition, we comprehensively compare the clinical and prognostic indexes among COVID-19 patients, patients with non-COVID-19 pneumonia, and healthy people during disease onset and the entire hospital stay. We built a computational model to predict prognosis at COVID-19 disease onset.

## Materials and Methods

### Participant Enrollment and Experimental Design

The National Health Commission approved this multicenter retrospective study in China. Each participating patient was provided written informed consent. A total of 110 domestic and 238 imported COVID-19 patients that were consecutively enrolled from January 21 to December 27, 2020, in 18 hospitals across 11 cities in Inner Mongolia. All enrolled patients were hospitalized, and COVID-19 infection was confirmed by the real-time reverse transcriptase-polymerase chain reaction (RT-PCR) test.

### Data Collection

For both 79 imported and 158 domestic COVID-19 patients, we obtained clinical data, including epidemiology, incubation time, symptoms, underlying comorbidities, therapeutic schedules, and examination results on 55 biochemical indices. In addition, we collected RT-PCR nucleic acid detection at nasopharyngeal swab, oropharyngeal swab, feces, sputum, and anal swab across multiple time points during disease onset and hospitalization for 197 patients with COVID-19 whose SARS-CoV-2 detection records were complete. The biochemical indices included blood routine, blood biochemistry, four coagulation, D-dimer, and blood gas, among others, all of which were measured approximately every 2 days.

According to “novel coronavirus infected pneumonia treatment scheme-Eighth edition” issued by the National Health Commission of the People’s Republic of China, the condition of a patient was diagnosed as one of the following four types:

(a) Mild: clinical symptoms are mild, and manifestations of pneumonia were not found on imaging;
(b) Moderate: exhibiting fever and respiratory symptoms, and radiographic evidence of pneumonia;
(c) Severe: satisfying any of the following: (1) shortness of breath, a respiratory rate of more than 30 breaths per minute; (2) peripheral blood oxygen saturation was less than 93% in resting-state; (3) PaO_2_/FiO_2_ 300 mmHg; and (4) severity of clinical symptoms was progressively increasing, and pulmonary imaging indicated that the lesion progression was more significant than 50% within 24–48 h; and
(d) Critical: satisfying any of the following: (1) respiratory failure occurs, and mechanical ventilation support is required; (2) shock; and (3) complicated with vital organ failure requires intensive care unit (ICU) treatment.

To compare COVID-19 patients with healthy people and patients with non-COVID-19 pneumonia (caused by other bacteria or viruses instead of SARS-CoV-2), the biochemistry indices of 131 matched healthy persons and 37 non-COVID-19 pneumonia were retrieved from hospitals in Inner Mongolia. Common pneumonia (non-covid-19 pneumonia) is usually divided into community-acquired pneumonia (CAP), hospital-acquired pneumonia, acute radiation pneumonia, ventilator-associated pneumonia, etc. “Non-Covid-19 pneumonia” in our study refers to CAP patients who meet one of (a) ∼ (d), and both (e) and (f), excluding pulmonary tuberculosis, tumor, non-infectious interstitial lung disease, pulmonary edema, pulmonary atelectasis, pulmonary embolism, eosinophilic pulmonary infiltration, pulmonary vasculitis. The criteria of Non-COVID-19 pneumonia were as follows: (a) Newly occurred cough, expectoration; or worsened chronic respiratory symptoms accompanied by purulent sputum; (b) Fever; (c) Signs of pulmonary consolidation and/or rales; (d) In peripheral blood, white blood cell count is higher than 10×10^9^/L or less than 4×10^9^/L. The cell nuclei shift to the left or not; (e) The results of the chest X-ray present emerging schistose, patchy infiltrative shadows or interstitial changes, with or without pleural effusion; (f) Negative results of COVID-19 nucleic acid test for at least five times.

### Statistical Analysis

We performed Fisher’s exact test to compare the COVID-19 detection rate between different sampling locations (e.g., feces vs. nasal swab and feces vs. pharyngeal swab). The student’s *t*-test was applied to assess differential biochemical indices between several different groups, including (1) mild vs. moderate COVID-19 patients, (2) imported vs. indigenous COVID-19 patients, and (3) COVID-19 patients vs. non-COVID-19 pneumonia and healthy people. The *P*-value was adjusted using the Bonferroni method for multiple testing when applicable. A test was significant if its *P*-value (adjusted *P*-value) was less than or equal to 0.05.R.

### Machine-Learning Models to Predict Mild and Moderate COVID-19 Patients

We adopted five distinct algorithms: NuSVC, Logistic RegressionCV, RidgeClassifierCV, RandomForestClassifier, and GaussianProcessClassifier (scikit-learn libraries in Python), to predict mild patients from moderate ones using the biochemical indices. Specifically, we utilized Python scikit-learn library function KNNImputer to fill in the missing values of features. We used a tenfold cross-validation scheme for prognostic prediction. For each fold, we calculated a probability for each patient in the validation dataset (10%) using coefficient estimates from the training dataset (90%). Then the predicted probability of each patient was then integrated into a whole dataset, based on which Receiver operating characteristic curves (ROC) and confusion matrix were drawn. We evaluated the prognostic model with three evaluation metrics, including the area under the receiver operating characteristic (AUROC), sensitivity, and specificity.

## Results

### Demographic and Clinical Characteristics

We enrolled 237 patients with COVID-19, 37 patients with non-COVID-19 pneumonia, and 131 healthy people for the clinical research related to COVID-19 disease. The demographic and clinical characteristics of the COVID-19 patients are summarized in Table 1. Specifically, 79 domestic COVID-19 patients with different severity of disease (mild, *n* = 11; moderate, *n* = 64; critical, *n* = 4; severe, *n* = 0) were recruited from 17 hospitals of 11 cities in the Inner Mongolia autonomous region of China. There were close family relations among 54 cases out of the 79 patients, verifying that COVID-19 was transmitted in a human-to-human pattern. The 158 imported COVID-19 patients include 59 mild, 96 moderate, and three serious cases, all imported into Inner Mongolia autonomous region from 16 different countries and regions. The percentage of mild cases from imported COVID-19 patients (37.3%) was far higher than that of the domestic ones (13.9%). Except for one critical patient who died, all of the other patients (236) recovered successfully from COVID-19. Age was significantly associated with the severity of COVID-19 (Spearman correlation 0.38 with *p*-value 1.091e-09). The median incubation period in the domestic group was 8 days (interquartile range, 4–11 days) compared with 5 days (interquartile range, 2–7 days) in the imported group. The incubation period was less than a week for approximately 50 and 75% of the domestic and imported patients, respectively. More than 5% of the patients, however, had an incubation period longer than 2 weeks, indicating that some people who were quarantined for 2 weeks still presented the risk of transmitting COVID-19. The median number of hospital stays for domestic patients was 22 days (interquartile range, 18–27 days) compared with 17 days (interquartile range, 15–25 days) for the imported population. Domestic patients with COVID-19 required approximately 5 more days of hospitalization than that of the imported patients with the same level of disease severity.

**TABLE 1:** Demographic and clinical characteristics of domestic and imported patients with COVID-19 with different severity.

Characteristic	Domestic				Imported			
	All (*n* = 79)	Mild (*n* = 11)	Moderate (*n* = 64)	Critical (*n* = 4)	All (*n* = 158)	Mild (*n* = 59)	Moderate (*n* = 96)	Serious (*n* = 3)
**Male, *n* (%)**	42 (53.2)	7 (63.6)	36 (56.3)	1 (25.0)	84 (53.2)	30 (50.8)	53 (55.2)	1 (33.3)
**Age, median (IQR), years**	45 (35–56)	37 (27–46)	45 (35–56)	69 (58–78)	36 (25–47)	25 (23–39)	40 (30–48)	69 (67–70)
Distribution Age—*n* (%)								
0–14 years	1 (1.2)	0 (0.0)	1 (1.6)	0 (0.0)	1 (0.6)	1 (1.7)	0 (0.0)	0 (0.0)
15–49 years	45 (57.0)	9 (81.8)	36 (56.3)	1 (25.0)	131 (82.9)	50 (84.7)	81 (84.4)	0 (0.0)
50–64 years	20 (25.3)	1 (9.1)	18 (28.1)	0 (0.0)	20 (12.7)	6 (10.2)	13 (13.5)	1 (33.3)
=65 years	13 (16.5)	1 (9.1)	9 (14.1)	3 (75)	6 (3.8)	2 (3.4)	2 (2.1)	2 (66.7)
**Incubation, median (IQR)-days**	8 (4–11)	8 (4–11)	8 (4–11)	3 (2–7)	5 (2–7)	4 (2–7)	5 (2–8)	13 (11–17)
Distribution incubation-*n* (%)								
0–7 days	26 (47.3)	4 (50.0)	20 (45.5)	2 (66.7)	123 (77.8)	51 (86.4)	72 (75.0)	0 (0.0)
8–14 days	27 (49.1)	3 (37.5)	23 (52.3)	1 (33.3)	26 (16.5)	6 (10.2)	18 (18.8)	2 (66.7)
= 15 days	2 (3.6)	1 (12.5)	1 (2.3)	0 (0.0)	9 (5.7)	2 (3.4)	6 (6.3)	1 (33.3)
NA*^a^*	24	3	20	1				
**Hospital stays, median-days**	22 (18–27)	22 (12–25)	22 (18–27)	33 (25–38)	17 (15–25)	16 (15–20)	18 (16–26)	29 (25–45)
Distribution hospital stays-*n* (%)								
0–14 day	11 (13.9)	4 (36.4)	7 (10.9)	0 (0.0)	19 (12.0)	13 (22.0)	6 (6.3)	0 (0.0)
15–21 day	24 (30.4)	1 (9.1)	22 (34.4)	1 (**??**)	90 (57.0)	33 (55.9)	56 (58.3)	1 (33.3)
22–30 day	33 (41.8)	4 (36.4)	29 (45.3)	0 (0.0)	30 (19.0)	8 (13.6)	21 (21.9)	1 (33.3)
= 31 day	11 (13.9)	2 (18.2)	6 (9.4)	3 (75.0)	19 (12.0)	5 (8.5)	13 (13.5)	1 (33.3)

*^a^NA means the data of incubation is not available for some COVID-19 patients.*

Of 79 domestic and 158 imported patients with COVID-19, the most common symptoms were fever (57.1/25.9%), cough (71.4/29.7%), and expectoration (42.9/15.5%) (Supplementary Table 1). Gastrointestinal symptoms such as dyspnea, nausea, and vomiting were also observed in patients with COVID-19 enrolled in this study. Notably, the percentage of domestic patients with COVID-19 showing typical symptoms was higher than that of the imported cases, even during the same periods of the disease.

The medication used to treat COVID-19 patients followed the guideline published by the National Health Committee, while some medicine varied with the individual course, which was the main reason for the difference in medication administered for the different types of COVID-19. All patients but one recovered and were discharged after hospitalization. The clinical treatment and outcomes are summarized in Supplementary Table 2. Generally, antibiotic medicine was used more often for the patients who progressed. The domestic patients more accepted bifidobacterium than the imported ones; the opposite trend was seen for Lianhuaqingwen. Chinese Mongolian medicine was widely used for both domestic and imported patients. Globulin was more frequently dosed in moderate and critical domestic cases than in moderate and critical imported cases. The prescription of Alpha interferon for the domestic patients was less common than for the imported ones, suggesting that most of the first group of patients visiting the doctors were mainly domestic patients, and the features of the pathogen were not yet identified at that time. Thus, the use of Alpha interferon was based on the experience of the doctors. Accumulating evidence showed that the Alpha interferon could work, and the percentage of Alpha interferon use rose accordingly, as did the use of Chloroquine medication. Note that the psychological situation had a tight relationship with the effects of the medicine. First, the more knowledge background of COVID-19 or a particular drug the patients had, the higher the variation observed in the treatment effect. Second, the more worried the patients were, the less effective the medication was for patients with the same types of COVID-19, including patients of the same sex, age range, and health history. Regretfully, neither a questionnaire nor a detailed recording of the psychological situation was provided to investigate this phenomenon further. In some cases, however, proper psychological interference was beneficial for recovery.

### COVID-19 RNA Dynamic Detection in Different Sampling Parts of the Body

A total of 2,944 samples tested for SARS-CoV-2 from 197 individuals were obtained during the hospitalization. Generally, compared with the higher individual positive detection with oropharynx/nasopharynx swabs, fecal swabs’ overall sensitivity dropped to 34.83% (62/178) (Supplementary Table 3). The cohort indicated that roughly two-thirds of COVID-19 patients might not have the SARS-CoV-2 virus in their feces.

The samples were divided into two parts according to the hospitalization period to study and compare the sensitivity of SARS-CoV-2 detection of different sampling anatomic sites in different disease stages. Samples collected during the first 7 days of hospitalization were defined as the early stage infectious type, and the subsequently collected samples were named the late-stage infectious type. The positive detection of SARS-CoV-2 in anatomic sites nasopharyngeal and oropharyngeal showed discrepancies, especially in the early stage (Table 2 and Supplementary Table 4). The positive detection with nasopharyngeal swabs was significantly higher than that of oropharyngeal swabs (Table 2).

**TABLE 2:** Statistically significant analysis of different COVID-19 positive detectable rate between feces and oropharynx/nasopharynx swab in the early and late period of infection.

	Stage	Sampling sites	Positive	Negative	Odds ratio	*P*-value*^c^*
Oropharynx vs. Nasopharynx	Early period of infection^a^	Oropharynx	80	114	0.62	**0.0251^d^**
		Nasopharynx	103	91		
	Late period of infection^b^	Oropharynx	73	620	1.02	0.9303
		Nasopharynx	72	622		
Oropharynx vs. feces	Early period of infection^a^	Oropharynx	19	23	0.75	0.6627
		Feces	22	20		
	Late period of infection^b^	Oropharynx	52	211	0.44	**2.2080e-05^d^**
		Feces	116	208		
Nasopharynx vs. feces	Early period of infection^a^	Nasopharynx	32	15	2.4	0.0598
		Feces	22	25		
	Late period of infection^b^	Nasopharynx	38	179	0.32	**2.875e-07^d^**
		Feces	87	131		

*^a^The early period of infection means 0–7 days since hospitalization.*

*^b^The late period of infection means > 7 days since hospitalization.*

*^c^Fisher’s exact test was used to compare the positive rate, and statistical significance is indicated by the P-value.*

*^d^Bold value represents statistically significant P-value < 0.05.*

A comparative analysis of the SARS-CoV-2 tests between oropharyngeal/nasopharyngeal swabs and feces specimens was performed for the patients who showed positive detection of SARS-CoV-2 at least once during the hospitalization process. The positive detection rate of SARS-CoV-2 in feces was significantly higher than in the oropharynx swabs (*P* = 2.2080e-05) and nasopharynx swabs (*P* = 2.8750e-07) for the late-stage infectious cases (Table 2 and Supplementary Table 4).

Notably, of 85 SARS-CoV-2 detect individuals, 17 cases were rebounding due to the defect in detection technology or increased viral load probably. One severe COVID-19 patient was positive in a feces swab, whereas negative in the oropharynx and oropharynx swab. This scenario lasted for more than ten days.

### Comparative Analysis of Biochemical Indices Among Different Groups

Fifty-five biochemical indices were performed comparing domestic and imported COVID-19 patients for the mild and moderate groups. Of these 55 biochemical indices, only the level of lymphocyte number (LYM_N), thrombin time (TT), and fibrinogen (FIB) in whole blood showed a significant difference with a *P*-value less than 0.001 (Bonferroni corrected *p*-value < 0.05) between imported and domestic patients with COVID-19 (Supplementary Figure 1). The LYM_N in imported patients with COVID-19 was statistically higher than in the domestic patients.

**FIGURE 1:**
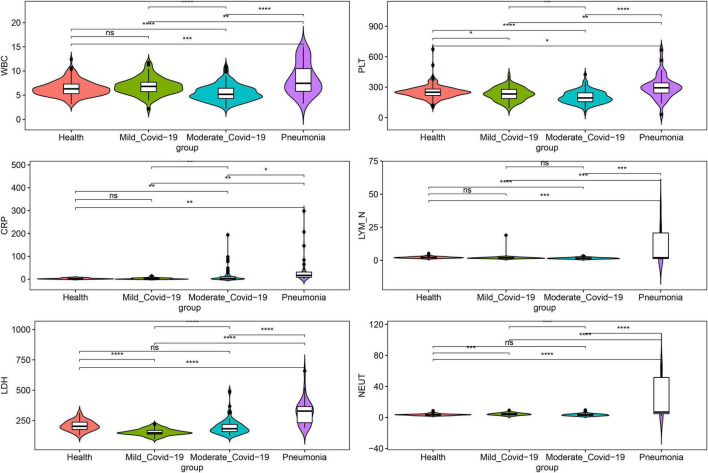
Comparison of six biochemical indices among the healthy, mild COVID-19, moderate COVID-19, and non-COVID-19 pneumonia groups. LDH, lactate dehydrogenase, PLT, platelet count, NEUT, neutrophilic granulocyte count, WBC, white blood cell count, CRP, C-reactive protein, LYM_N, lymphocyte count, Imp_mild, imported mild COVID-19, Imp_moderate, imported moderate COVID-19, Ind_mild domestic mild COVID-19, Ind_moderate domestic moderate COVID-19. ns *P* > 0.05, *P < 0.05, **P < 0.01, ***P < 0.001, ****P < 0.0001 (Student’s *t*-test).

We compared six biochemical indices, including lactate dehydrogenase (LDH), platelet counts (PLT), neutrophilic granulocyte count (NEUT), white blood cell count (WBC), C-reactive protein (CRP), and LYM_N, among healthy, mild-type COVID-19, moderate-type COVID-19, and non-COVID-19 pneumonia groups (Figure 1). Non-COVID-19 pneumonia patients showed a significantly higher WBC, LYM_N, and PLT than all other groups. Moderate COVID-19 patients presented significantly higher CRP levels than the healthy group, while Mild COVID-19 patients showed no significant difference. In contrast, CRP in the non-COVID-19 pneumonia group was the highest among all groups. Surprisingly, LDH significantly decreased in mild COVID-19 patients compared with healthy people, while the moderate group showed no significant change; furthermore, it was extremely high in patients with non-COVID-19 pneumonia. In addition, in comparison with healthy controls, COVID-19 patients showed a substantial decrease in five blood indicators, including absolute eosinophils count, percentage of eosinophils, absolute basophil count, total proteins, hemoglobin concentration, and neutrophil count percentage increased significantly (Supplementary Figure 2).

### Machine-Learning Models Predicting the Clinical Outcome Based on Biochemical Indices on Admission Day

**FIGURE 2:**
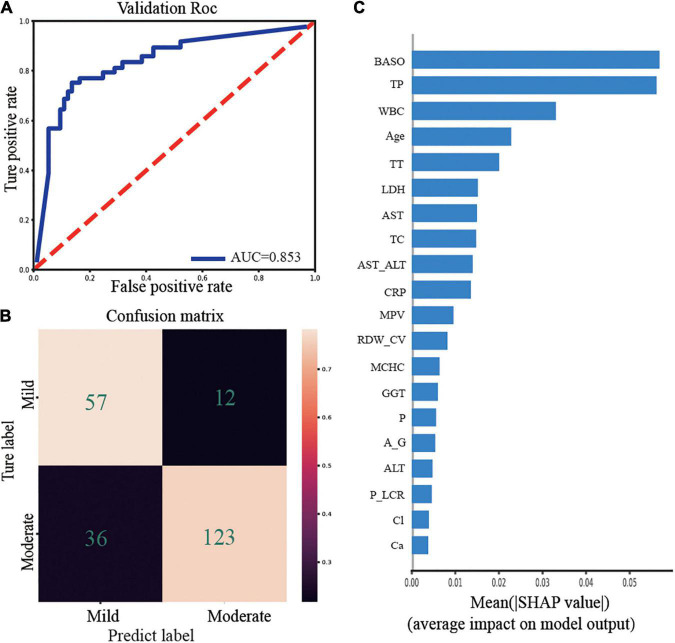
Prediction classifier model evaluation and feature importance. **(A)** ROC curve for predicting mild and moderate COVID-19. **(B)** Confusion matrix of Random forest classifier of mild and moderate with hematology indices. **(C)** Barplot of feature importance for the prediction model. BASO, basophil cell count, TP, total protein, WBC, white blood cell, Age, the age of patients, TT, thrombin time, LDH, lactic dehydrogenase, AST, glutamic oxalacetic transaminase, TC, total cholesterol, AST_ALT, AST/ALT, CRP, C-reactive protein, MPV, mean platelet volume, RDW_CV, red blood cell distribution width, MCHC, mean corpuscular hemoglobin concentration, GGT, glutamyl transpeptidase, P, phosphorus in the blood, A_G, albumin/globulin, ALT, alanine transaminase, P_LCR, platelet-large cell ratio, Cl, the chlorine in the blood, Ca, calcium.

We established a model to predict the clinical outcomes of the mild and moderate COVID-19, excluding the severe and critical COVID-19 types because of their small sample size. We applied five algorithms, including NuSVC, LogisticRegressionCV, RidgeClassifierCV, RandomForestClassifier, and GaussianProcessClassifier, to build prediction models with 55 biochemical indices and demographic characteristics of mild and moderate COVID-19 patients on admission day. RandomForestClassifier performed the best, and the result of ROC curve analysis is shown in Figure 2A. The area under the curve (AUC) for the prediction of mild and moderate COVID-19 was 0.853 in the tenfold cross-validation test. Figure 2B shows the confusion matrix for the mild- and moderate-type classification in the testing set. The medians of the model evaluation parameters sensitivity and specificity were 82.61 and 77.36%, respectively.

Feature importance estimates are crucial for both the interpretability and robustness of prediction models. Hence, to interpret the built prediction model, we adopted the Shapley Additive exPlanations (SHAP) technique, which uses the Shapley values to explain the contribution of each feature to the prediction (22). Figure 2C depicts a bar plot of SHAP values that integrates the feature importance with the average impact on the prediction model. BASO contributed the most to the predictive model in classifying the mild and moderate types of COVID-19 patients. The contribution of TP to the prediction model is almost as significant as that of WBC.

### Dynamic Changes of Blood Biochemical Indices in COVID-19 Patients

**FIGURE 3:**
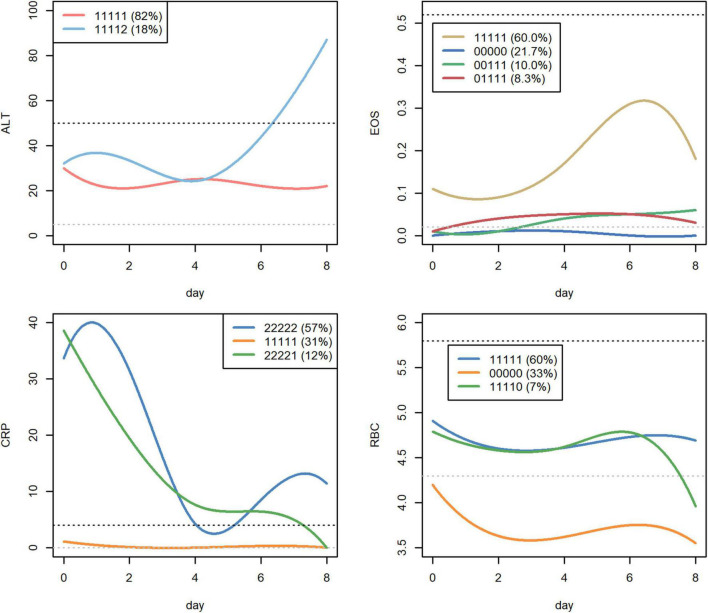
Dynamic detection of biochemistry indices in COVID-19 patients. The first five hospital records of biochemistry indices, including ALT, CRP, EOS, and RBC, were shown for dynamic analysis. The numbers 0, 1, and 2 in the dynamic change pattern mean that the biochemical indices of COVID-19 patients are lower than the standard value, within the healthy range, and higher than the expected value, respectively.

The dynamic change pattern of four biochemical indices is shown in Figure 3. Eighteen percent of COVID-19 patients presented higher serum ALT than the normal range. CRP was elevated in more than 69% of COVID-19 patients; furthermore, 57% of patients maintained a higher CRP level than the normal range throughout the hospital stay. Almost 40% of COVID-19 patients presented with a lower RBC level than the normal range, and 33% of patients maintained a lower RBC level than the normal range during the whole hospital stay.

The percentage of patients with COVID-19 showing an elevated ALT level was significantly higher in the moderate patients than in the mild patients. In contrast, the percentage of patients with COVID-19 showing decreased EOS and RBC levels were significantly higher in moderate patients than in mild patients (Supplementary Table 5).

## Discussion

Our study conducted a comprehensive comparison of laboratory test results that provided a practical approach to evaluate the potential COVID-19 prognosis. Our analysis also provides insights into the COVID-19 sample collection. We discovered that the positive detection rate of COVID-19 was significantly higher in feces than in oropharynx-nasopharynx swabs in the late stage of the disease.

### Comparison of Clinical Characteristics in Domestic and Imported COVID-19 Patients

The median age of imported patients was 9 years younger than that of domestic patients, probably because the population of returnees was generally young. Statistical results showed that the number of hospital stay days in the imported patients was 5 days shorter than in the domestic patients. Furthermore, the imported patients had milder symptoms than the domestic patients did. This may have been because imported patients were younger and thus recovered faster or because the drug treatment used for these patients was more effective than for domestic patients. The median incubation period was only 4 days (interquartile range, 2–7) in earlier COVID-19 patients in China (5, 23). In contrast, the median incubation period was 8 days (interquartile range, 4–11), and the most prolonged incubation period was 18 days for native COVID-19 patients in Inner Mongolia.

Furthermore, more than 5% of the imported COVID-19 patients had an incubation period longer than 15 days. Consequently, people from high-risk areas remained at risk of developing and spreading COVID-19 after 14 days of isolation. Notably, close contact with a COVID-19 patient isolated for 14 days with a negative nucleic acid test triggered an epidemic of COVID-19 in Beijing, China. On November 26, 2020, he entered Indonesia and was in close contact, on the same flight, and in the same row seat, with an imported case from Indonesia reported by Fujian Province. After 14 days of isolation in Fujian, he arrived in Beijing on December 10 and went to the Shunyi district. On December 26, the patient’s nucleic acid test for COVID-19 was negative, but the serum IgM antibody was positive. On December 27, he was found positive for a nucleic acid test in his living and working environment (the 196th press conference of Beijing epidemic prevention and control). Therefore, we strongly suggest that people from high-risk areas be quarantined for 21 days or longer to reduce potential hazards more efficiently.

Imported and domestic patients with COVID-19 showed significantly different at three biochemical indices, including LYM_N, TT, and FIB. A possible explanation is that the virus strain that invaded the imported patients may differ from the domestic one. The virulence between the imported and the domestic one may also be different. Considering the longer TT and lower FIB in the imported patients compared with the domestic cases, it could be inferred that the hepatocytes in the imported patients were more damaged than in the domestic patients.

### Comparison of Clinical Characteristics in Mild and Moderate COVID-19 Patients

In the moderate-type group, there were more patients with abnormally high ALT than those in the mild-type group. ALT is one of the essential catalysts in human metabolism that mainly exists in the hepatocytes. When certain viruses or bacteria infection happens, it can induce liver inflammation. The infected hepatocytes lose their integrity, and ALT leaks out of the damaged hepatocytes, resulting in the ALT elevation in the blood test (24). Thus, the extent of hepatocellular damage in the moderate patients was much more severe than the situation in the mild patients. The severity of the disease is positively associated with the severity of hepatocellular damage.

More patients with moderate-type COVID-19 had fewer EOSs than those in the mild-type group. EOS, a kind of WBCs, plays an essential role in the immune responses and the allergic process (25, 26). There are abundant thick and evenly disposed of granules in the cytoplasm. These granules are released because EOSs can easily break when they fight against an invader, and the granules released could induce tissue injury and promote inflammation (27, 28). Two possible reasons might explain moderate-type COVID-19 had fewer EOSs than mild-type patients: first, the severity of the moderate type led to more consumption of EOSs; second, when the infection of COVID-19 progressed to a certain extent, such as from mild to moderate, the normal function of the bone marrow might have been inhibited (29–31), leading to the decreased production of EOSs from the bone marrow. In contrast, the hematopoietic functional inhibition also could cause less RBC production. It seemed that such inhibition of the RBC was positively related to the severity of COVID-19. Note that RBC also possessed an immune role (32). Thus, the decreased number of RBC in the moderate-type group of COVID-19 might reflect the battle between the virus and the immune system.

LDH levels in the COVID-19 mild group were significantly decreased compared with those in COVID-19 moderate patients and the healthy people. LDH is a cytosolic enzyme and metalloprotein containing zinc ions, broadly existing in the myocardium, liver, kidney, skeletal muscle, lung, etc. Since LDH participates in anaerobic glycolysis and gluconeogenesis, its changes reflect the body’s metabolism. Therefore, abnormal LDH levels can be seen in various pathological processes. In most scenarios, LDH is elevated, and the underlying mechanism has been well elucidated. However, it had been claimed that the decrease of LDH had no clinical significance until it was found to decrease in psychological depression (33). There still has been reported that LDH might be dropped within people who presented the depressive symptoms when working in stressful environments (34). In our study, we have already noticed the patients’ psychological situation and the corresponding psychotherapy could influence the recovery. Thus, the depressive mood occurred to a different extent in the COVID-19 patients, regardless of the patients. Although there was no sign of pneumonia in the mild COVID-19 group, the patients were worried and depressed, leading to reduced LDH compared to other groups, even the health group. As far as the moderate group is concerned, LDH reduced because of the depressive mood; however, the pathological infiltration and damage happened, resulting in the leak of LDH from the damaged cells and the increased LDH. The balance between the two sides presented a similar LDH level as that in the health group. Logically, the level of LDH was higher in the critical patients. In non-COVID-19 pneumonia, the abnormally high LDH level was consistent with the results from other research (35).

### The Role of the Dynamic Monitoring of Biochemical Indices in the Clinical Treatment of COVID-19 Patients

In addition to CT scan, active supervision of the biochemistry indices is one of the essential methods used to observe patients with COVID-19 and evaluate the body’s immune system and multi-organ functions. Based on the dynamic change of these indices, medication was adjusted correctly. Close attention should be given to the combination of indices to evaluate the situation (e.g., take a blood routine test), and the absolute value of the number of lymphocytes should be combined with the neutrophil-to-lymphocyte ratio (NLR) and lymphocyte-to-C-reactive protein ratio (LCR) to provide a preliminary impression of the virus infection. In addition, dynamically supervision of routine blood tests, including NLR, LCR, and other components in the blood and their ratio or proportion to each, should be undertaken to monitor the working situation of the bone marrow and the body’s immune system. As for the enzymological indices to monitor liver function, all the enzymes should be assayed and integrated to have a panorama. To date, it is widely accepted that COVID-19 could bring unimaginable damage to essential organs in both the short term and the long term. Its influence is not evident in the long run and will continue to be studied in the coming years. Given this reality, dynamic monitoring of biochemistry indices is the least costly and undoubtedly has been ideal for treatment during hospitalization and in the clinical follow-up process.

### The Positive Detection Rate of COVID-19 in Feces Was Higher Than Oropharynx-Nasopharyngeal Swabs

COVID-19 patients typically exhibit a wide range of symptoms, including fever, cough, expectoration, headache, and sore throat (5, 7, 36). Additionally, gastrointestinal symptoms, such as diarrhea, nausea and vomiting, loss of appetite, and abdominal pain, have been reported in the presented study and multiple other studies (7, 37, 38), indicating that COVID-19 infects the lungs as the intestines and liver (39–41). A review of 48 independent studies revealed that approximately 11 and 12% of COVID-19 patients exhibited diarrhea, vomiting, and nausea, respectively (42). In contrast, in this study, only 7 and 3.2% of imported COVID-19 patients showed diarrhea, vomiting, and nausea, respectively. However, more than 30% of COVID-19 patients were found SARS-CoV-2 RNA in their feces. Therefore, the SARS-CoV-2 can appear in the intestines of patients without gastrointestinal symptoms, implying the importance of using feces for virus testing for COVID-19 patients being discharged.

Interestingly, we found higher SARS-CoV-2 detection rates with feces swab than oropharynx-nasopharynx swab in cases in the late stages of the disease. Some COVID-19 patients remained positive for SARS-CoV-2 in the feces after the oropharynx and nasopharynx swabs turned negative, which is consistent with a previous study (43). A significant difference in SARS-CoV-2 detection between feces and the oropharynx-nasopharynx swabs was identified. Of critical concern in evaluating the risk of a fecal-oral transmission pathway for COVID-19 is the degree of infectivity of fecal-derived virus particles.

## Data Availability Statement

The original contributions presented in the study are included in the article/Supplementary Material, further inquiries can be directed to the corresponding author/s.

## Ethics Statement

The studies involving human participants were reviewed and approved by the National Health Commission. The patients/participants provided their written informed consent to participate in this study.

## Author Contributions

DS and HZ conceived the project. LY and AW implemented the experiments, and analyzed the data. WJ, CY, FG, XF, and HW prepared the data. TL and GT performed literature search. AW wrote the manuscript. All authors approved the final manuscript.

## Conflict of Interest

AW, TL, and GT were employed by the company Geneis (Beijing) Co., Ltd. The remaining authors declare that the research was conducted in the absence of any commercial or financial relationships that could be construed as a potential conflict of interest.

## Publisher’s Note

All claims expressed in this article are solely those of the authors and do not necessarily represent those of their affiliated organizations, or those of the publisher, the editors and the reviewers. Any product that may be evaluated in this article, or claim that may be made by its manufacturer, is not guaranteed or endorsed by the publisher.

## Supplementary Material

Supplementary Figure 1 Violin plot of the three statistically significant different biochemical indices between domestic and imported COVID-19 patients. LYM_N, TT, and FIB on the Y-axis represent absolute monocyte, prothrombin time, and fibrinogen, respectively. Imp_Mild, Imp_Moderate, Ind_Mild, and Ind_Moderate on the X-axis represent imported mild-type COVID-19 patients group, imported moderate-type COVID-19 patients group, domestic mild-type COVID-19 patients group, and domestic moderate-type group, respectively. ^ns^*P* > 0.05, ***P*<0.01, ****P*<0.001 (Student’s *t*-test).

Supplementary Figure 2 Comparison of six biochemical indices among the healthy, mild COVID-19, and moderate COVID-19 groups. EOS, absolute eosinophils count; EO, percentage of eosinophils; BASO, absolute basophil count; TP, total proteins; HGB, hemoglobin concentration; NEU_P, neutrophil count percentage; Mild_Covid-19, mild-type COVID-19; Moderate_Covid-19, moderate-type COVID-19. ^ns^*P* > 0.05, **P*< 0.05, ***P*< 0.01, ****P* < 0.001 (Student’s *t*-test).

Supplementary Table 1 Clinical symptoms of patients with COVID-19.

Supplementary Table 2 Clinical treatment of COVID-19 patients.

Supplementary Table 3 The positive rate of SARS-CoV-2 RNA detection in different sampling sites for COVID-19 patients.

Supplementary Table 4 The comparison of SARS-CoV-2 detection among different sampling sites.

Supplementary Table 5 Significant different dynamic change of biochemical indicators between Moderate- and Mild-COVID-19 patients.
