# Supplementary figures and images for: A Retrospective and Multicenter Study on COVID-19 in Inner Mongolia: Evaluating the Influence of Sampling Locations on Nucleic Acid Test and the Dynamics of Clinical and Prognostic Indexes
Source: Front Med (Lausanne). 2022 Mar 30;9:830484. doi: 10.3389/fmed.2022.830484 (PMC9007405; doi:10.3389/fmed.2022.830484)

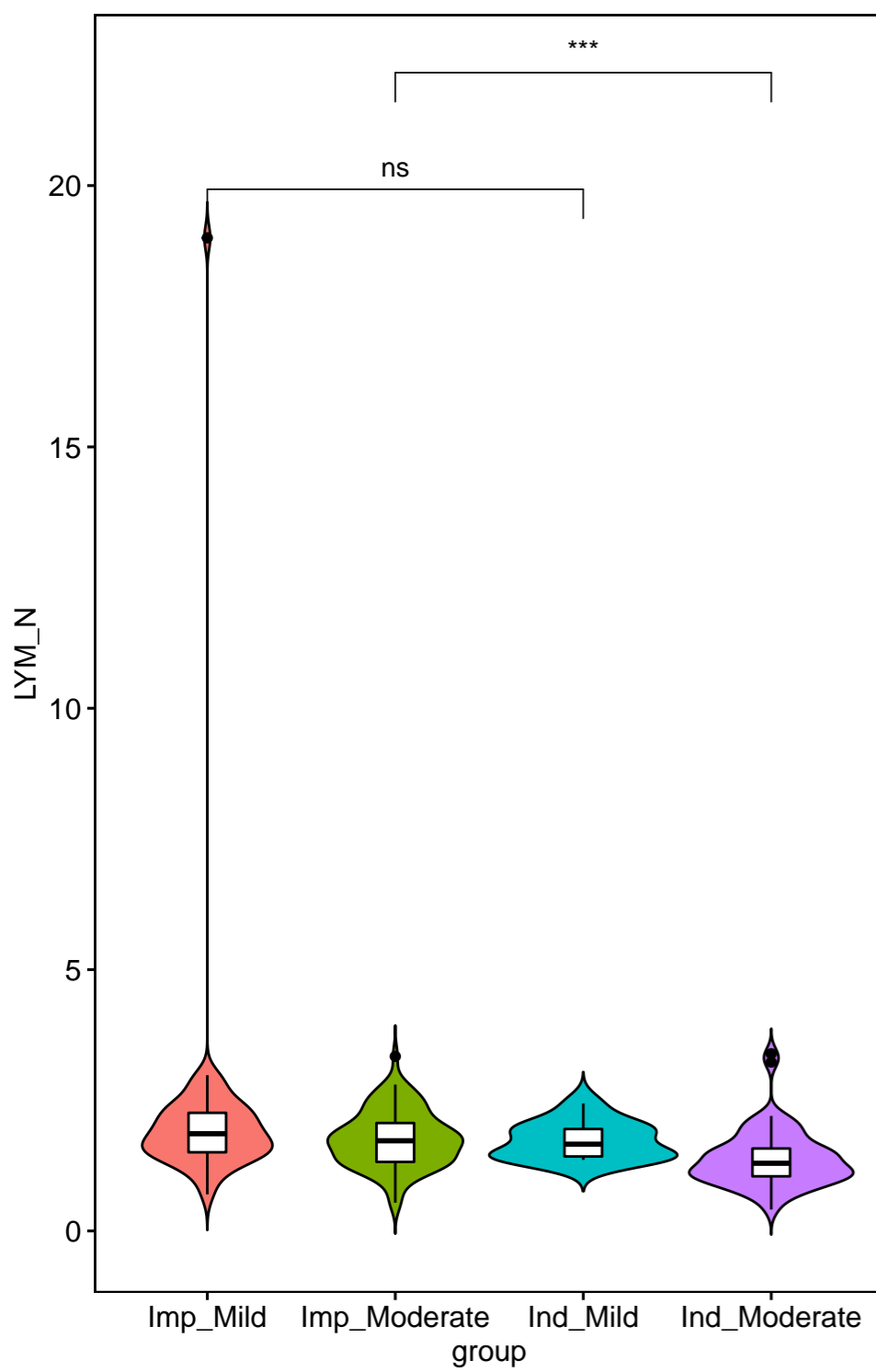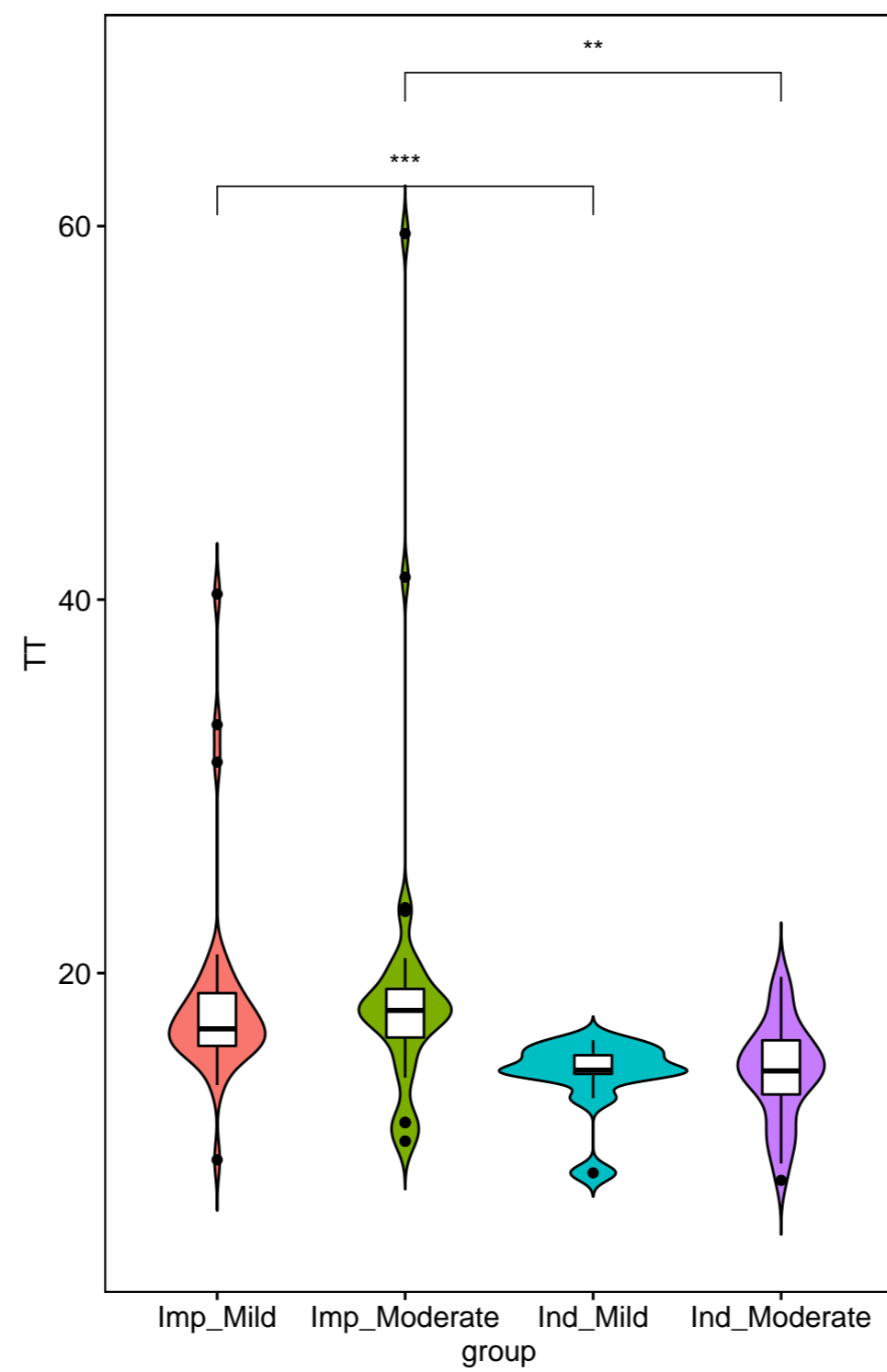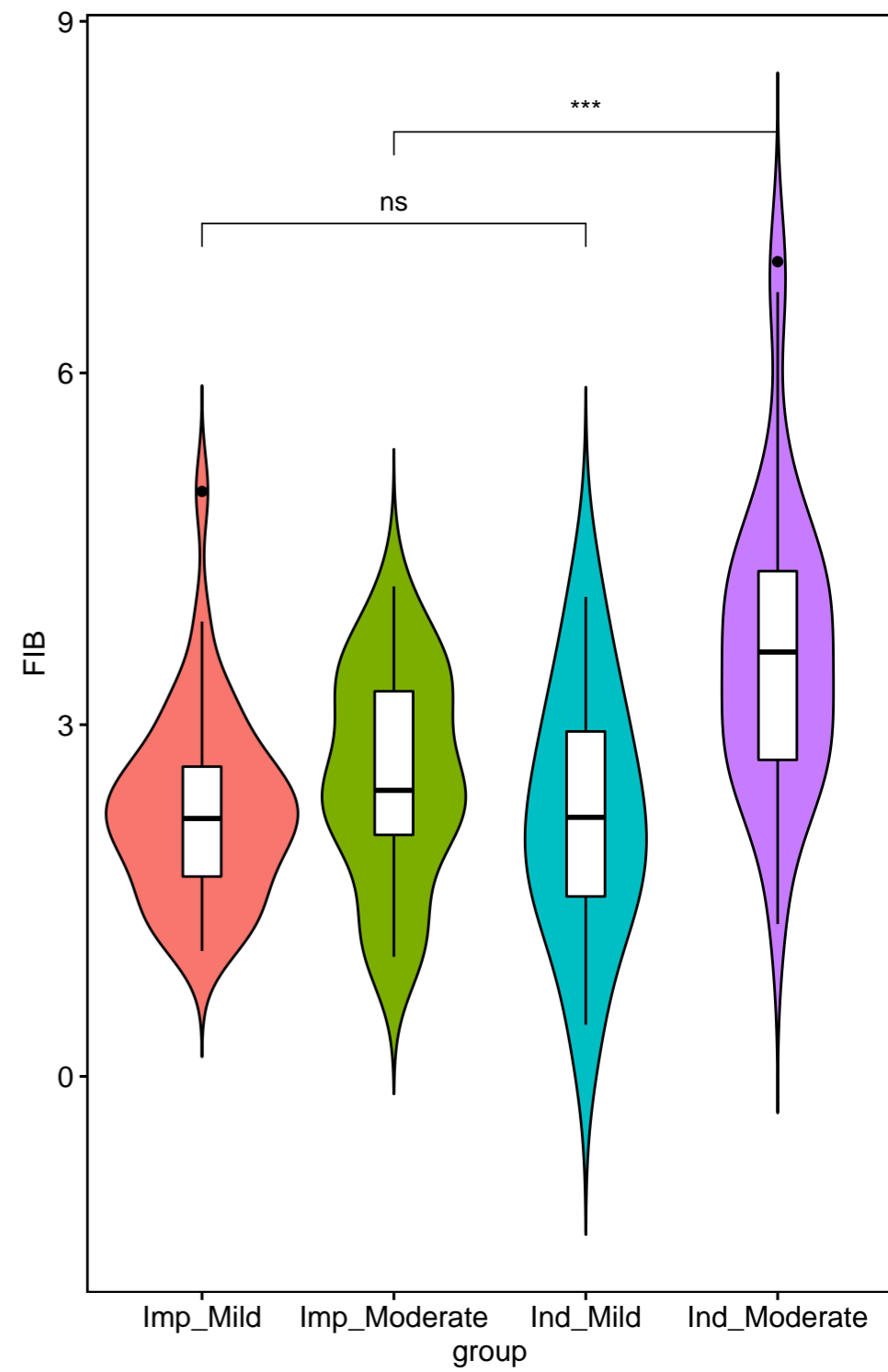

Supplement: Supplementary Figure 1 — Violin plot of the three statistically significant different biochemical indices between domestic and imported COVID-19 patients. LYM_N, TT, and FIB on the Y-axis represent absolute monocyte, prothrombin time, and fibrinogen, respectively. Imp_Mild, Imp_Moderate, Ind_Mild, and Ind_Moderate on the X-axis represent imported mild-type COVID-19 patients group, imported moderate-type COVID-19 patients group, domestic mild-type COVID-19 patients group, and domestic moderate-type group, respectively. nsP > 0.05, **P<0.01, ***P<0.001 (Student’s t-test). [file Image_1.pdf]

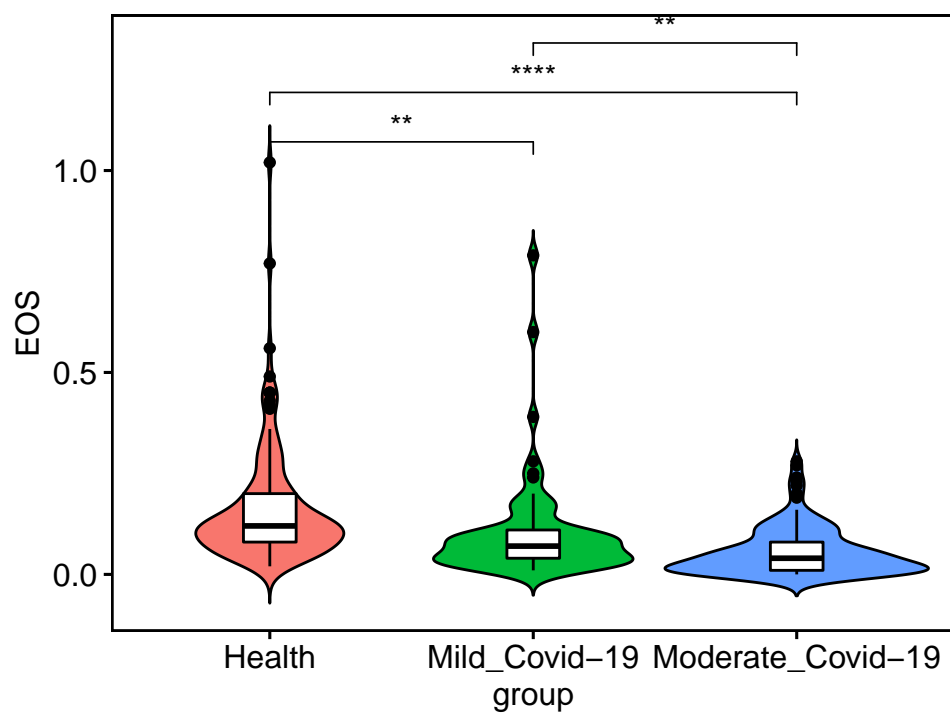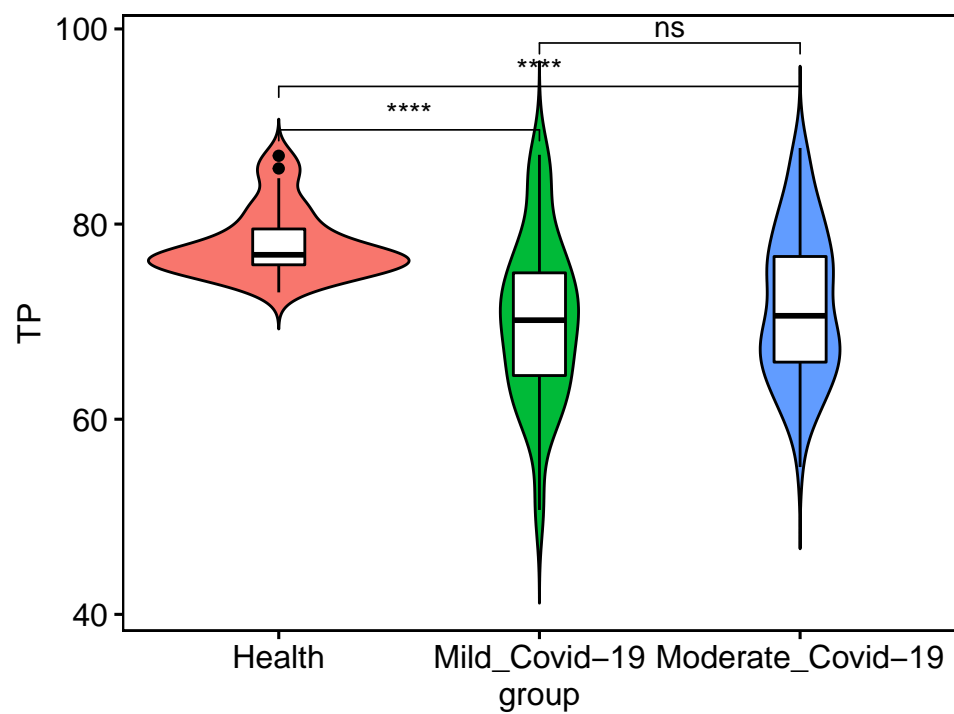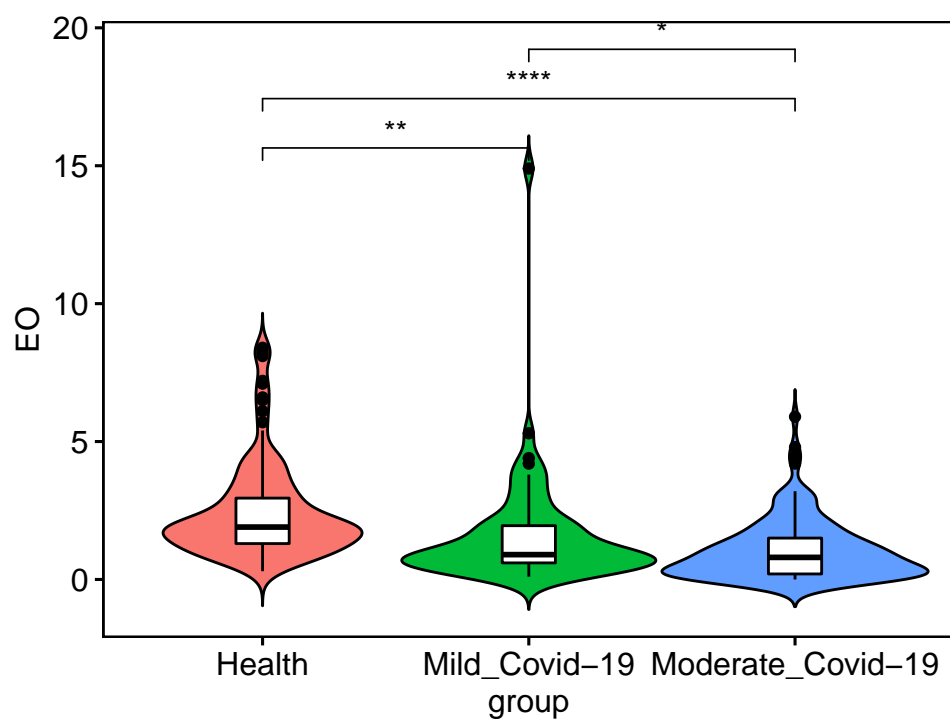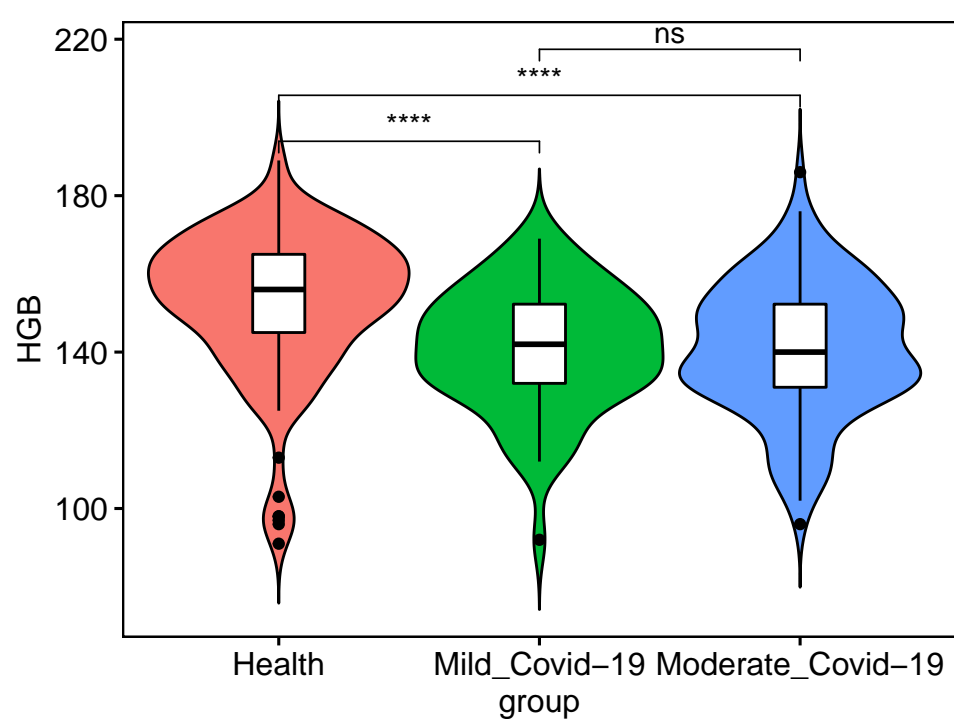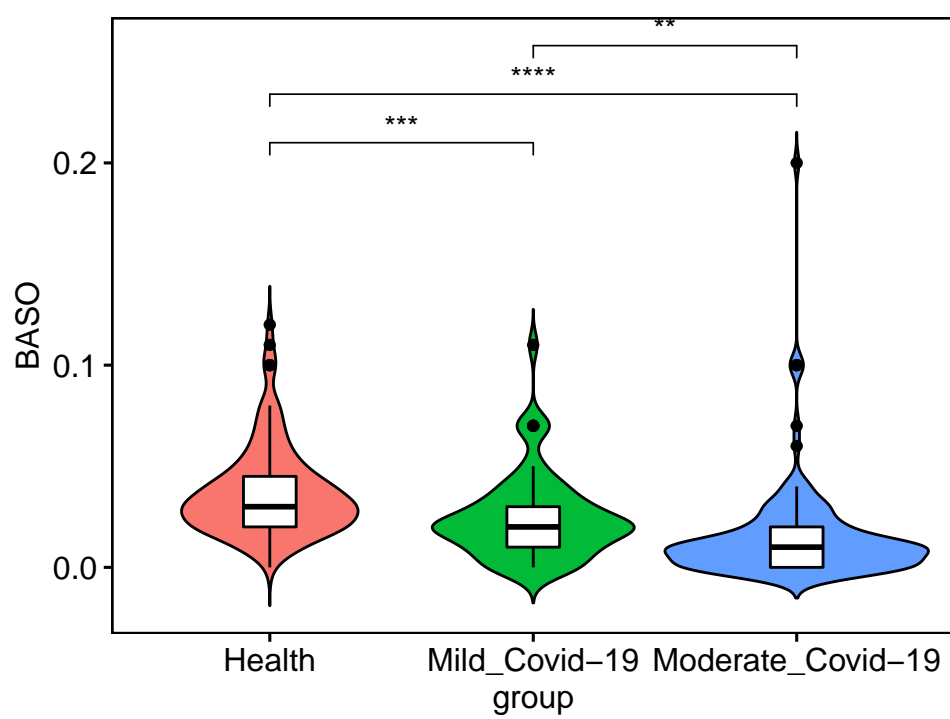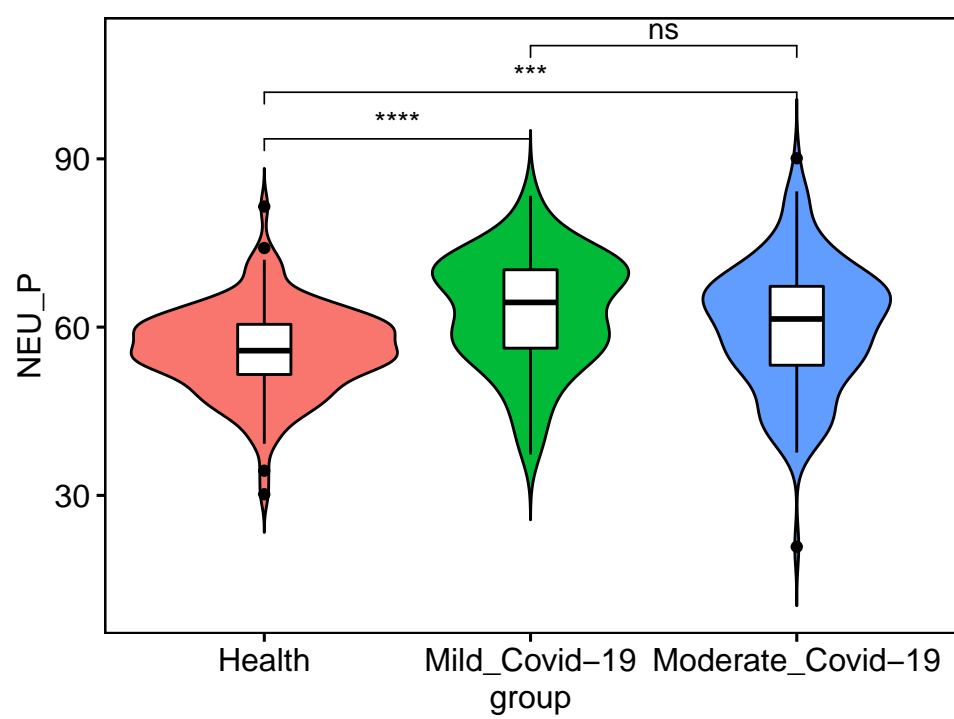

Supplement: Supplementary Figure 2 — Comparison of six biochemical indices among the healthy, mild COVID-19, and moderate COVID-19 groups. EOS, absolute eosinophils count; EO, percentage of eosinophils; BASO, absolute basophil count; TP, total proteins; HGB, hemoglobin concentration; NEU_P, neutrophil count percentage; Mild_Covid-19, mild-type COVID-19; Moderate_Covid-19, moderate-type COVID-19. nsP > 0.05, *P< 0.05, **P< 0.01, ***P < 0.001 (Student’s t-test). [file Image_2.pdf]
