# Supplementary material for: A Retrospective and Multicenter Study on COVID-19 in Inner Mongolia: Evaluating the Influence of Sampling Locations on Nucleic Acid Test and the Dynamics of Clinical and Prognostic Indexes
Source: Front Med (Lausanne). 2022 Mar 30;9:830484. doi: 10.3389/fmed.2022.830484 (PMC9007405; doi:10.3389/fmed.2022.830484)
Supplement: Supplementary Table 1 — Clinical symptoms of patients with COVID-19. [file Table_1.DOCX]

**Table S1.** Clinical symptoms of patients with COVID-19

|  | **Domestic** | | | | **Imported** | | | |
| --- | --- | --- | --- | --- | --- | --- | --- | --- |
| **Symptoms** | All  (79) | Mild  (n=11) | Moderate  (n=64) | Critical  (n=4) | All  (158) | Mild  (n=59) | Moderate  (n=96) | Serious  (n=3) |
| Fever | 44(57.1) | 3(27.3) | 38(61.3) | 3(75) | 41(25.9) | 12(20.3) | 27(28.1) | 2(66.7) |
| Cough | 55(71.4) | 7(63.6) | 45(72.6) | 3(75) | 47(29.7) | 10(16.9) | 35(36.5) | 2(66.7) |
| Expectoration | 33(42.9) | 4(36.4) | 26(41.9) | 3(75) | 24(15.5) | 4(7.1) | 18(18.8) | 2(66.7) |
| Dyspnea | 15(19.7) | 0(0) | 12(19.7) | 3(75) | 11(7.0) | 3(5.1) | 7(7.3) | 1(33.3) |
| Fatigue | 29(38.2) | 1(9.1) | 27(44.3) | 1(25) | 12(7.6) | 7(11.9) | 4(4.2) | 1(33.3) |
| Muscle pain | 11(14.5) | 0(0) | 11(18.0) | 0(0) | 4(2.5) | 2(3.4) | 2(2.1) | 0(0) |
| Headache | 11(14.5) | 0(0) | 10(16.4) | 1(25) | 10(6.4) | 4(6.8) | 6(6.3) | 0(0) |
| Stuffy | 8(10.5) | 2(18.2) | 6(9.8) | 0(0) | 15(9.5) | 3(5.1) | 12(12.5) | 0(0) |
| Diarrhea | 16(20.8) | 2(18.2) | 13(21) | 1(25) | 11(7.0) | 3(5.2) | 8(8.3) | 0(0) |
| Nausea and vomiting | 5(9.6) | 0(0) | 5(12.2) | 0(0) | 5(3.2) | 2(3.4) | 3(3.2) | 0(0) |
| Sore throat | 12(15.8) | 1(9.1) | 10(16.4) | 1(25) | 29(18.4) | 7(11.9) | 22(22.9) | 0(0) |

**Table S2.** Clinical treatment of COVID-19 patients

| **Treatment** | **Domestic**  **(n=79)** | | | | **Imported**  **(n=158)** | | | |
| --- | --- | --- | --- | --- | --- | --- | --- | --- |
|  | **All**  **domestic** | **Mild**  **(n=11)** | **Moderate**  **(n=64)** | **Critical**  **(n=4)** | **All**  **imported** | **Mild**  **(n=59)** | **Moderate**  **(n=96)** | **Serious**  **(n=3)** |
| Alpha interferon | 35(44.3) | 6(54.5) | 25(39.0) | 4(100) | 137(86.7) | 49(83.1) | 85(88.5) | 3(100) |
| ntibiotic | 26(32.9) | 2(18.8) | 20(31.2) | 4(100) | 17(7.6) | 4(6.8) | 7(7.2) | 1(33.3) |
| Arbidol | 6(7.6) | 0(0) | 5(7.8) | 1(25) | 3(1.9) | 0(0.0) | 3(3.1) | 0(0.0) |
| Bifidobacterium | 42(53.2) | 5(45.5) | 33(51.6) | 4(100) | 30(19.0) | 10(16.9) | 18(18.8) | 2(66.7) |
| Chinese Mongolian  medicine | 42(54.4) | 6(54.5) | 33(51.6) | 4(100) | 102(64.6) | 43(72.9) | 56(58.3) | 3(100) |
| Chloroquine | 4(5.0) | 0(0) | 2(3.1) | 2(50) | 132(83.5) | 45(76.3) | 84(87.5) | 3(100) |
| Globulin | 17(21.5) | 0(0) | 14(21.9) | 3(75) | 6(3.8) | 0(0) | 5(5.2) | 1(33.3) |
| Lianhuaqingwen | 9(11.4) | 3(27.3) | 6(9.3) | 0(0) | 123(77.8) | 43(72.9) | 78(81.3) | 2(66.7) |
| Lopinavir | 30(38.0) | 5(45.5) | 22(34.4) | 3(75) | 42(26.6) | 23(39.0) | 16(16.7) | 3(100) |
| Methylprednisolone | 16(20.3) | 0(0) | 12(18.8) | 4(100) | 16(10.1) | 1(1.7) | 13(13.5) | 2(66.7) |
| Ribavirin | 6(7.6) | 0(0) | 5(7.8) | 1(25) | 1(0.6) | 0(0) | 1(1.0) | 0（0.0） |
| Thymosin | 12(15.2) | 3(27.3) | 8(12.5) | 1(25) | 11(7.0) | 1(1.7) | 8(8.3) | 2(66.7) |
| Xuebijing | 43(54.4) | 4(36.4) | 35(54.7) | 4(100) | 30(19.0) | 1(1.7) | 26(27.1) | 3(100) |
| **Clinical outcome** |  |  |  |  |  |  |  |  |
| Discharged | 78(98.7) | 11(100) | 64(100) | 3(75) | 158(100) | 59(100) | 96(100) | 3(100) |
| Died | 1(1.3) | 0(0) | 0(0 ) | 1(25) | 0(0) | 0(0) | 0(0) | 0(0) |

**Table S3**. The positive rate of SARS-CoV-2 RNA detection in different sampling sites for COVID-19 patients.

| Sampling sites | Positive | Negative | Positive rate (%) |
| --- | --- | --- | --- |
| Oropharynx | 166 | 28 | 85.57 |
| Nasopharynx | 113 | 78 | 59.16 |
| Feces | 62 | 116 | 34.83 |

**Table S4**. The comparison of SARS-CoV-2 detection among different sampling sites

| Oropharynx vs. Nasopharynx | | | |
| --- | --- | --- | --- |
| The early period of infection |  | Nasopharynx |  |
| (0-7 days since hospitalization) | Oropharynx | Negative | Positive |
|  | Negative | 83 | 31 |
|  | Positive | 8 | 72 |
| The late period of infection |  | Nasopharynx |  |
| (>7 days since hospitalization) | Oropharynx | Negative | Positive |
|  | Negative | 595 | 25 |
|  | Positive | 26 | 47 |
| Oropharynx vs Feces | | | |
|  |  | Feces |  |
| The early period of infection | Oropharynx | Negative | Positive |
| (0-7 days since hospitalization) | Negative | 15 | 7 |
|  | Positive | 4 | 15 |
|  |  | Feces |  |
| The late period of infection | Oropharynx | Negative | Positive |
| (>7 days since hospitalization) | Negative | 134 | 77 |
|  | Positive | 14 | 38 |
| Nasopharynx vs. Feces | | | |
|  |  | Feces |  |
| The early period of infection | Nasopharynx | Negative | Positive |
| (0-7 days since hospitalization) | Negative | 13 | 5 |
|  | Positive | 6 | 17 |
|  |  | Feces |  |
| The late period of infection | Nasopharynx | Negative | Positive |
| (>7 days since hospitalization) | Negative | 131 | 72 |
|  | Positive | 13 | 25 |

**Table S5.** Significant different dynamic change of biochemical indicators between Moderate- and Mild-COVID-19 patients

| **Indicator** | **Change** | **Moderate** | **Mild** | ***P*-value** |
| --- | --- | --- | --- | --- |
| ALT | Elevated | 43 | 8 | **0.044** |
|  | Normal | 26 | 13 |  |
| EOS | Decreased | 47 | 7 | **0.003** |
|  | Normal | 34 | 20 |  |
| RBC | Decreased | 60 | 8 | **0.001** |
|  | Normal | 24 | 19 |  |
